# Erbin: an important therapeutic target for blocking tumor metastasis

**DOI:** 10.3389/fphar.2024.1474798

**Published:** 2024-09-26

**Authors:** Tingting Qiu, Liquan Tan, Jialong Yan, Qunli Luo

**Affiliations:** ^1^ Department of hematopathology, The Affiliated Changsha Central Hospital, Hengyang Medical School, University of South China, Changsha, China; ^2^ Department of Nursing, The Affiliated Changsha Central Hospital, Hengyang Medical School, University of South China, Changsha, China; ^3^ Institute of Clinical Research, The Affiliated Nanhua Hospital, Hengyang Medical School, University of South China, Hengyang, China

**Keywords:** Erbin, tumor, metastasis, antitumor immunity, platelets

## Abstract

Erbin is an adapter protein that interacts with the v-erb-b2 avian erythroblastic leukemia viral oncogene homolog 2 (ERBB2) in epithelial cells. Erbin plays an important role in various signaling pathways, including cell proliferation, apoptosis, and autophagy. Additionally, Erbin is implicated in the pathogenesis and progression of sepsis and various cancers, including breast cancer, acute myeloid leukemia (AML), hepatocellular carcinoma (HCC), and colorectal cancer (CRC). A recent study shows that loss of Erbin increases the release of acyl-carnitine (Acar) through abolishing interaction with prothrombotic protein endothelial cell-specific adhesion molecule (ESAM), promotes mitochondrial oxidative phosphorylation in B cells, and ultimately suppresses lung metastasis of CRC. Accordingly, Erbin provides us with a new potential treatment for tumor metastasis.

## Introduction

Erbin is a member of the leucine-rich repeat and PDZ domain (LAP) family that acts as an adapter for the v-erb-b2 avian erythroblastic leukemia viral oncogene homolog 2 (ERBB2) in epithelia (Borg et al., 2000). Erbin’s PDZ domain binds to the C-terminal tail of the ERBB2 receptor (Santoni et al., 2020). Erbin interacts with the unphosphorylated form of the ERBB2 protein, regulating ERBB2 function and localization (Teyra et al., 2021). Erbin localizes at the basolateral membrane of polarized epithelial cells, as well as at post-synaptic densities where it binds to cell surface receptors (Santoni et al., 2020). Erbin is recognized as primarily localizing to adherens junctions and contributes to the preservation of the junction’s structural integrity in epithelial cells (Stevens et al., 2018). Erbin is found in most human tissues, such as the brain, liver, kidney, spleen, gut, and skeletal muscle (Matsushima et al., 2019). Erbin plays an important role in various signaling pathways and clinical diseases.

Erbin is involved in a range of diverse functions, including inflammation response, cell proliferation, differentiation, apoptosis, and autophagy (Jang et al., 2021). Erbin functions as a classical regulator of inflammation response due to its ability to bind specifically to intracellular receptor nucleotide-binding oligomerization domain-containing protein 2 (NOD2) and then upregulate proinflammatory cytokines (Droghini et al., 2022). The RAS-RAF-ERK pathway plays an important role in cell proliferation, differentiation, apoptosis, and stress response. When extracellular signals activate RAS, it combines with Erbin to form a complex that prevents RAS from activating RAF (Huang et al., 2003). Similarly, the Erbin-Merlin complex breaks the interaction between PAK2 and GTP-bound CDC42/RAC1, thereby inhibiting the transforming growth factor beta (TGF-β) pathway (Royo et al., 2020), which is involved in cell differentiation and apoptosis. Erbin deficiency causes excessive activation of autophagy, resulting in autophagy cell death (Larabi et al., 2020). Likewise, the accumulation of Erbin restrains RAF activation to prevent autophagy and senescence in KRAS proto-oncogene (Xie et al., 2015). Therefore, given the diverse functions of Erbin in various signaling pathways, it is suggested that Erbin may play a vital role in clinical disease.

Erbin is connected to various clinical diseases. For example, mosaic chromosomal alterations (mCAs) increase the incidence of Crohn’s disease (CD). Interestingly, a genome-wide association study shows that Erbin was substantially linked to mCAs in patients with CD (Kakuta et al., 2022). Similarly, Shen et al. (2018) reveal a crucial role of Erbin in the inhibition of excessive activation of autophagy and autophagic cell death, which gives rise to a new strategy for inflammatory bowel disease therapy. Meanwhile, differentially expressed genes (DEGs) revealed Erbin was deferentially expressed in autism spectrum disorder (Rahman et al., 2020). Moreover, Erbin deficiency in breast cancer cells led to resistance to targeted medicines such as trastuzumab and lapatinib and accelerated cancer invasion and metastasis (Liu D. et al., 2013). A somatic mutation of Erbin was also discovered in patients with metastatic cholangiocarcinoma, resulting in a CD4^+^ T cell-directed immune response and evident cancer regression (Tran et al., 2014). Mutations in the desmoglein-1 (DSG1) gene lead to the development of striate palmoplantar keratoderma (SPPK). Interestingly, silencing Erbin in keratinocytes disrupted cell differentiation, which recapitulates the phenotypes seen in SPPK patients carrying DSG1 mutations (Harmon et al., 2013). Additionally, Erbin inhibition enhances the NRG1-ErbB signaling pathway, resulting in the inhibition of apoptosis and subsequent repair of spinal cord injury in mice (Wu et al., 2019). That means Erbin has great promise in the treatment of spinal cord injury.

**FIGURE 1:**
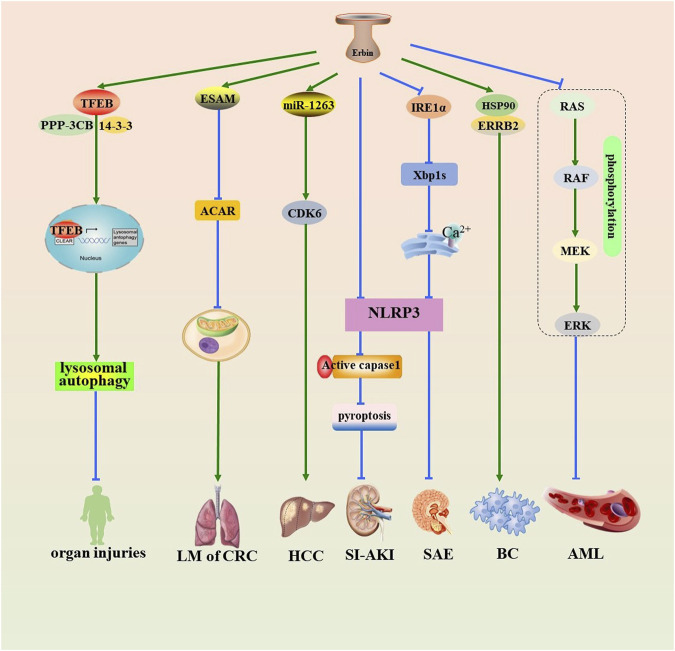
Role of Erbin in the pathogenesis of sepsis and various tumors. Erbin promotes lysosomal autophagy by interacting with TFEB and improving the stability of the TFEB-14-3-3 and TFEB-PPP3CB complexes, promoting the activation of TFEB and the nucleus translocation, which leads to alleviating organ injuries. Erbin inhibits Acar by promoting contact with the prothrombotic protein ESAM, thus alleviating the activity of the mitochondrial oxidative phosphorylation in B cells and suppressing lung metastasis. Erbin promotes the proliferation of HCC by serving on a sponge of miR-1263 that subsequently targets CDK6. Erbin can alleviate SI-AKI by inhibiting pyroptosis mediated by NLRP3/caspase-1 signaling. Erbin inhibits IRE1/Xbp1s pathway activity and reduces the endoplasmic reticulum Ca^2+^ influx to the cytoplasm. This inhibition restrains the downstream activation of NLRP3 inflammasomes and pyroptosis, thereby protecting against sepsis-associated encephalopathy. Erbin facilitates the interaction between ERRB2 and HSP90, thereby contributing to the initiation and progression of BC. Erbin inhibits the cell proliferation of AML cells by decreasing the phosphorylation levels of the RAS/RAF/MEK/ERK pathway. Erbin, Erbb2-interacting protein; TFEB, transcription factor EB; Acar, acetyl-carnitine; ESAM, endothelial cell-specific adhesion molecule; HCC, hepatocellular carcinoma; miR-1263, microRNA-1263; CDK6, cyclin-dependent kinase 6; SI-AKI, sepsis-induced acute kidney injury; NLRP3, NOD-like receptor thermal protein domain-associated protein 3; caspase-1, cysteine-requiring aspartate protease-1; IRE1α, inositol-requiring enzyme 1 alpha; Xbp1s, X-box binding protein 1; ERRB2, recombinant receptor tyrosine protein kinase erbB-2; HSP90, heat-shock protein90; BC, breast cancer; AML, acute myeloid leukemia.

Erbin is implicated in the pathogenesis and progression of sepsis and various cancers (Figure 1), including breast cancer, acute myeloid leukemia (AML), hepatocellular carcinoma (HCC), and colorectal cancer (CRC) (Figure 1). Autophagy-lysosome pathway (ALP) dysfunction is considered a potential toxic mechanism of sepsis. Transcription factor EB (TFEB) is one of the MiT-TFE family members, regulating the expression of lysosomal and autophagy-related genes. Erbin promotes lysosomal biogenesis and autophagy by interacting with TFEB and improving the stability of the TFEB-14-3-3 and TFEB-PPP3CB complexes, promoting the activation of TFEB and the nucleus translocation, which leads to alleviating inflammatory responses and organ injuries of sepsis (Fang et al., 2023). Analogously, Erbin knockdown can aggravate sepsis-induced acute kidney injury (SI-AKI) by promoting pyroptosis mediated by NOD-like receptor thermal protein domain associated protein 3 (NLRP3) inflammasome/caspase-1 signaling (Liu Y. et al., 2023). Erbin inhibits inositol-requiring enzyme 1 alpha (IRE1α)/X-box binding protein 1 (Xbp1s) pathway activity and restricts the endoplasmic reticulum (ER) Ca^2+^ influx to the cytoplasm. This inhibition restrains the activation of NLRP3 inflammasomes and microglial pyroptosis, thereby mitigating the release of abundant inflammatory cytokines and ultimately protecting against sepsis-associated encephalopathy (Jing et al., 2022).

Erbin facilitates the interaction between ERRB2 and heat-shock protein90 (HSP90), a crucial protein for maintaining ERRB2 stability, thereby contributing to the initiation and progression of breast cancer (Tao et al., 2014). The RAS/RAF/MEK/ERK pathway is one of the mitogen-activated protein kinase (MAPK) pathways, which are closely related to the development of various cancers (Song et al., 2023). Erbin inhibits the cell proliferation of AML cells by decreasing the phosphorylation levels of the RAS/RAF/MEK/ERK pathway (Zheng et al., 2019). The downregulation of NF-κB inhibitor α(IκBα) promoted HCC tumorigenesis via upregulation of NF-κB-mediated Erbin expression (Tan et al., 2020). Erbin promotes the proliferation of HCC by serving on a sponge of microRNA-1263 (miR-1263), which subsequently targets cyclin-dependent kinase 6 (CDK6) (Yang et al., 2022). Erbin promotes the progression of CRC through the miR-125a-5p/miR-138-5p/4EBP-1axis activated cap-independent HIF-1α translation (Chen et al., 2020). Meanwhile, Erbin is a potential target for the treatment of lung metastasis of CRC (Shen et al., 2021). Recently, Zhang et al. (2024) demonstrated that an Erbin-mitochondria axis in platelets/megakaryocytes (MKs), which suppress B-cell-mediated antitumor immunity, which suggests a promising method of treating metastasis.

In order to clarify the role of Erbin to CRC metastasis, Zhang et al. (2024) observed a significant increase in platelet count among CRC patients with metastasis as well as high Erbin expression in platelets from CRC patients with metastasis. Afterward, they established a clinically relevant mice model with complete deletion of mice (Erbin^−/−^) and MK-specific Erbin-deficient mice (referred to as cKO). A reduction in platelet counts and stronger aggregation capability in Erbin^−/−^ or cKO mice was observed through platelet aggregation assay. Meanwhile, cKO mice exhibited significantly suppressed lung metastasis of CRC. As we know, the primary mechanism of human attack on tumor cells is through cellular immunity, which is predominantly mediated by T cells. The immunosuppressive molecule programmed death 1 (PD1), when combined with programmed cell death 1 ligand 1(PDL1) on tumor cells, significantly impairs cellular immune function and thereby promotes the survival of tumor cells (Gao et al., 2024). Surprisingly, the numbers of PD1^+^ or PDL1^+^ were significantly lower in cKO mice. These results suggest that Erbin knockout in platelets/MKs inhibits the metastasis of CRC in mice and enhances the aggregation of platelets in the lung through the downregulation of PD1/PDL1.

To further elucidate the contribution of Erbin-deficient platelets/MKs during antitumor immunity, they employed adoptive cell transfer therapy (ACT) by transferring MKs or platelets isolated from Erbin cKO mice to WT mice, respectively. Using ACT with platelets from cKO mice resulted in significant inhibition of CRC lung metastasis and exhaustion of T cells. Furthermore, the number of PD1^+^ plasma cells and PD1^+^CD8^+^ T cells in the lung was notably reduced in ACT-treated WT mice compared with untreated WT mice. Subsequently, Zhang et al. (Shen et al., 2021) isolated B cells from Erbin cKO mice, significantly inhibiting CRC metastasis. Additionally, WT mice receiving B cells isolated from cKO mice exhibited a notable decrease in proportions of PD1^+^ CD4^+^ or CD8^+^ T cells and a significant increase in cytotoxic CD4^+^ or CD8^+^ T cells, particularly those secreting perforin and interferon (IFN). That means the antitumor effect of Erbin-deficient platelets on CD8^+^ T cells is dependent on B cells.

The tandem mass tag-based quantitative proteomics technique and immunofluorescence were used to analyze differential proteins in platelets isolated from cKO mice and WT mice. Results showed endothelial cell-specific adhesion molecule (ESAM), a key protein mediating cell adhesion and scaffold reconstruction, was expressed at a low level in Erbin-deficient platelets. Interestingly, as crucial coenzymes in redox reactions, nicotinamide adenine dinucleotide (NAD^+^) and its reduced state (NADH) are indispensable for fundamental mitochondrial metabolic pathways. As expected, the ratio of NAD^+^/NADH in Erbin-deficient platelets was significantly higher than that in WT platelets. In order to identify the key metabolites involved in Erbin-regulated mitochondria, they used off-target metabolomic sequencing and discovered that Erbin-deficient platelets had higher acyl-carnitine (Acar) concentrations. Erbin-deficient platelets exhibit increased mitochondrial oxidative phosphorylation and produce more lipid metabolites, such as Acar, by interacting and downregulating the prothrombotic protein ESAM.

Palmitoyl-L-carnitine (PLC) was used to mimic the effects of Acar in an *in vitro* experiment. The enzyme activities of the complex proteins of the mitochondrial electron transport chain were significantly increased in B cells from cKO or WT mice after Acar-PLC treatment. Analysis of the NAD^+^/NADH ratio showed that Acar-PLC treatment enhanced the oxidative phosphorylation of mitochondria in B cells isolated from cKO or WT mice. Furthermore, endogenous immunoprecipitation revealed that E3 ubiquitin ligase FBXO38 interacted with PD1 in B cells. PLC enhanced the acetylation of FBXO38 and promoted the binding of FBXO38 protein and PD1 protein in B cells. Acar epigenetically enhances the activity of the mitochondrial electron transport chain complex and mitochondrial oxidative phosphorylation in B cells by H3K27ac, promotes the acetylation of the E3 ligase FBXO38 to ubiquitinate, and degrades the PD1 protein. Furthermore, they developed a nanovesicle system that encapsulated Erbin small-interfering RNA (siErbin) and delivered siErbin into platelets/MKs. Targeting Erbin in platelets/MKs *in vivo* successfully suppressed lung metastases of CRC in WT mice. Targeting Erbin in platelets/MKs greatly increased the numbers of plasma cells secreting IFNg or perforin and dramatically decreased the number of PD1^+^ plasma cells and PD1^+^CD8^+^ T cells in the lung of mice.

Therefore, the Erbin-mitochondria axis is a potential strategy for the treatment of CRC metastasis. Fortunately, many inhibitors of Erbin have been identified, including lipopolysaccharides (LPSs)/nigericin, miR-23c, SAG, and Erbin-3451-RNAi. LPSs are the main component of the cell wall of Gram-negative bacteria. Nigericin is a K^+^, H^+^-ionophore with antibiotic, antimalarial, and antiviral potency. The levels of murine Erbin were downregulated following LPS/nigericin stimulation (Jing et al., 2022). miR-23c is a member of the microRNAs (miRNAs) family that can attenuate the expression of Erbin, thus suppress tumor growth and induces apoptosis (Zhang et al., 2018). SAG, an essential component of the SKP1, Cullins, and F-box proteins (SCF) E3 ligase, promotes Erbin ubiquitylation and degradation, thereby causing ROS accumulation to trigger autophagy and senescence (Xie et al., 2015). Similarly, the inhibitory effect of RNAi interference on Erbin is particularly significant, especially Erbin-3461-RNAi (Wu et al., 2019). These inhibitors may open new avenues for therapeutic intervention in tumor metastasis.

In summary, Erbin knockout in platelets/MKs suppressed lung metastasis. Mechanically, Erbin-deficient platelets exhibit increased mitochondrial oxidative phosphorylation and release Acar by inhibiting contact with the prothrombotic protein ESAM. Acar increased the activity of the mitochondrial electron transport chain complex and mitochondrial oxidative phosphorylation in B cells by epigenetically acetylating H3K27. Collectively, the Erbin-mitochondria axis in platelets/MKs reduces B cell-mediated antitumor immunity, implying a new method for treating metastasis.

## Data Availability

The original contributions presented in the study are included in the article/supplementary material; further inquiries can be directed to the corresponding author.
